# Cognitive and contextual factors modulating grammar learning at older ages

**DOI:** 10.3389/fnagi.2022.943392

**Published:** 2022-08-31

**Authors:** Marta Rivera, Daniela Paolieri, Antonio Iniesta, Teresa Bajo

**Affiliations:** Department of Experimental Psychology, Mind, Brain, and Behavior Research Center (CIMCYC), University of Granada, Granada, Spain

**Keywords:** second language learning, aging, individual differences, context of learning, proactive control

## Abstract

Second language learning has been shown more difficult for older than younger adults, however, the research trying to identify the sources of difficulty and possible modulating factors is scarce. Extrinsic (learning condition and complexity) and intrinsic factors (executive control) have been related to L2-grammar learning in younger adults. In the present study, we aim to assess whether extrinsic and intrinsic factors are also modulating grammar learning in older adults. We compared the learning performance of younger and older adults in a L2 learning task. 162 Spanish native-speakers (81 young) learnt *Japañol* (Japanese syntaxis and Spanish lexicon) in either an intentional (metalinguistic explanation) or an incidental (comprehension of sentences) context. The complexity of the sentences was also manipulated by introducing (or not) a subordinate clause. Individual differences in proactivity were measured with the AX-CPT task. After the learning phase, participants performed a Grammatical Judgment Task where they answered if the presented sentences were grammatically correct. No differences between older and younger adults were found. Overall, better results were found for the intentional-condition than for the incidental-condition. A significant interaction between learning context and the proactivity index in the AX-CPT task showed that more proactive participants were better when learning in the incidental-condition. These results suggest that both extrinsic and intrinsic factors are important during language learning and that they equally affect younger and older adults.

## Introduction

Learning a second language (L2) late in life has been shown to be a tool to access new social and cultural challenges in a globalized world (Pot et al., 2019) as well as a source of cognitive enhancement (Bubbico et al., 2019). The benefits associated to language learning at older ages have been broadly studied, both after a brief exposure to a new language (Wong et al., 2019; see Nilsson et al., 2021 for a review) and after a lifetime of speaking more than one language (Bialystok et al., 2016; but see Papageorgiou et al., 2019 for a different view). However, the actual process of learning late in life and the differences to language learning in younger adults have been less investigated.

Aging is assumed to be related to cognitive decline (Park et al., 2002; Craik and Grady, 2009), due to structural changes in the brain (see Park and Reuter-Lorenz, 2008 for a review). Age-related impairments have been observed in executive functioning (EF, Craik and Bialystok, 2006; see Verssimo et al., 2021, for nuances), working memory (WM; Salthouse, 2009; Pliatsikas et al., 2019), declarative memory (Ullman, 2016; Ward et al., 2020) and intentional/explicit learning, where there is an intention to learn something (see Hedden and Gabrieli, 2004 for a review). However, the decline in incidental/implicit learning, that is learning without intention, is not so well documented (Ward et al., 2020). The existing studies have shown that implicit learning is less susceptible to aging than explicit learning (Ristin-Kaufmann and Gullberg, 2014); not only when motor skills are involved, but also during language learning (structural priming; Hardy et al., 2019; speech production; Muylle et al., 2021), and it is better preserved for older adults with greater cognitive abilities (Howard and Howard, 2013; Fu et al., 2020; Ward et al., 2020). Hence, the goal of this study is to investigate the possible role of cognitive abilities in intentional/explicit and incidental/implicit learning for older adults with a specifically focus on executive functions (EF), since previous studies with younger adults have shown that better EF skills might facilitate language learning (Kapa and Colombo, 2014).

EF refer to a domain-general set of mechanisms that control cognition and action to attain a specific goal (Miyake and Friedman, 2012). An interesting theoretical framework in the context of aging (Braver and Barch, 2002) and language use (Pérez et al., 2018) is the Dual Mechanisms of Cognitive Control (DMC, Braver, 2012). According to this framework, two different cognitive control modes (proactive and reactive), as part of the EF set of mechanisms, may be put into work in different situations. Proactive control refers to anticipatory goal selection that minimizes interference before a distracting event occurs (Braver, 2012) and it is highly related to working memory (Unsworth et al., 2009). Reactive control can be understood as a late correction mechanism operating in a bottom-up manner to avoid interference once it has occurred. The interaction between these two control modes is dynamic so that people might differ in their use of the two control modes, and some situations may favor one mode over the other (Mäki-Marttunen et al., 2019). Crucially, the AX-CPT (AX-Continuous Performance task; Ophir et al., 2009) has been designed to assess individual differences in the relative balance between proactive and reactive control. In this task, participants need to answer to a pair of cue/probe. Participants answer “yes” when the cue is an A, and the probe is an X (AX trials) and “no” in any other situation. Proactive and reactive control preference is calculated thanks to the combination of the AY (A-Cue, Y-probe) and BX (B-cue, X-probe) trials. For instance, BX trials can benefit from proactive but not reactive control and the opposite is found for AY trials, that benefit from reactive but not proactive control.

Differences in proactivity/reactivity as measured by the AX-CPT task have been related to age (Braver and Barch, 2002), and therefore, they might also be related to older and younger differences in grammar learning. Results comparing younger and older performance in the AX-CPT task have shown that younger adults typically rely on proactive control more than older adults who tend to use more reactive control strategies (Braver et al., 2009). This pattern has been attributed to the high cognitive demand associated to proactive control (Braver, 2012) and the difficulties of older adults to maintain the relevant contextual information needed to reduce contextual interference in advance (Braver and West, 2008; Xiang et al., 2016). Therefore, contrary to younger adults, older adults may compensate their reduced ability for proactive strategies by using more reactive strategies when learning a new language. Additional studies have found that older adults with a cognitively active daily live (reading, playing instruments, or having high education for example), hence with high cognitive reserve, might use the same strategies than younger adults both cognitively (Stern, 2009) and during learning (Gajewski et al., 2020). As mentioned, proactive control has been related to language use (Pérez et al., 2018) and to language learning (Rivera et al., under review), and, it is, then, possible, that variations in cognitive control may play a key role predicting grammar learning in incidental/implicit and intentional/explicit learning contexts. If this was the case, the pattern of individual differences in grammar leaning might differ for younger and older adults. However, no study to date has tested the influence of proactive/reactive strategies during grammar learning in older adults.

Many of the studies comparing incidental/implicit and intentional/explicit language learning in young adults have used semi-artificial and artificial grammar learning (AGL) where participants are assumed to learn grammatical rules implicitly or explicitly. During AGL, participants are exposed, and sometimes instructed, to memorize letter sequences that follow a particular rule (Reber, 1967). After the learning phase, participants are informed that the sequences followed rules and they are asked to classify new letter sequences as grammatical or ungrammatical based on this information. In many cases, participants are not informed or aware of the regularities conforming the grammatical rule while being exposed to them (implicit/incidental learning), whereas in intentional/explicit conditions, they might be informed about the existence of a rule or even explicitly informed of the specific nature of the rule and asked to learn it for further testing. Overall, studies using semi-artificial and AGL (see Goo et al., 2015 for a meta-analysis) report that participants (usually young adults) obtain better results from intentional than incidental learning, suggesting that metalinguistic knowledge enhances learning.

Interestingly, experiments comparing older and younger adults in artificial grammatical learning have shown that older adults show impaired performance during intentional learning relative to the younger adults, however, these differences are reduced or not evident during incidental learning (Kürten et al., 2012). For instance, Midford and Kirsner (2005) created four conditions in which the complexity of the artificial grammar and the explicit or implicit nature of the instruction was varied. Across experiments these conditions were tested in older and younger adults: (a) in the first experiment, the presented letter strings conformed a complex grammar system, and participants did not receive instructions or explanations about the rules; (b) in the second experiment, the same complex grammar system was used but participants received detailed instructions to understand the grammar structure; (c) in the third experiment, the presented strings conformed a simple grammar system, and participants did not receive instructions about the rules; and (d) in the last experiment, the strings conforming the simple grammar system were presented, and participants received instruction with detailed explanation about the grammatical system. After the training phase, all participants performed a test judging whether the presented sequences were or not grammatically correct, and finally, they were asked to report the strategies that they might have used during the test (memory/guessing). Overall, younger adults were more accurate than older adults. Additionally, they found an interaction between age and grammatical rule-complexity, indicating that younger adults were more accurate when learning an easy grammar rule than older adults, however, these differences were not evident for difficult grammar learning. An additional interaction between grammatical rule-complexity and instructions also indicated better performance when explicit instructions were given for simple grammar learning than when participants were not given learning instructions (implicit), however, these differences were not found for complex grammar learning.

The similar performance of older and younger participants in the implicit learning of complex grammatical rules suggests that implicit learning might be preserved in older adults (Midford and Kirsner, 2005). Additionally, analyses of self-reported strategies indicated that both groups used explicit strategies (memory) when the rule was easy or explicitly presented. However, they tended to use implicit strategies (guessing) when the rule was complex or implicitly presented. This pattern also suggests that older adults are less effective than younger adults in their use of explicit strategies, but that implicit strategies might be efficiently used by the older adults since learning differences with the younger adults where not evident in conditions where these strategies were required (implicit complex conditions) and used (Midford and Kirsner, 2005). More recent data also indicate that older adults seem to relay more on incidental than intentional learning strategies (see Wagnon et al., 2019 for a review). Since older adults have a notable decay in declarative memory, the differences between younger and older adults in language learning might also be related to the cognitive resources available to the participants, so that age related impairments in cognitive resources might reduce the efficacy of intentional strategies in explicit/intentional learning conditions (Ingvalson et al., 2017).

Individual differences in cognitive abilities might, then, be an important factor modulating grammar learning in incidental and intentional conditions. Individual differences in cognitive processes (Luque and Morgan-Short, 2021), including WM (Faretta-Stutenberg and Morgan-Short, 2018), declarative/procedural learning/memory skills (Morgan-Short et al., 2012; Fu and Li, 2021) and EFs (Kapa and Colombo, 2014; Rivera et al., under review) have been related to language learning in young adults. Since there have been found age related differences in the cognitive control (Braver and West, 2008), in the present study we will focus on proactive cognitive control as a possible source of individual differences that might underlie the age-related differences in grammar learning.

The main goal of this experiment was to investigate whether differences between younger and older adults would be observed during intentional and incidental learning of a semi-artificial grammar. Additionally, we wanted to explore the influence of extrinsic (instruction and difficulty of the grammatical rules) and intrinsic factors (individual differences in proactive/reactive control) in the learning process and whether the influence of these factors change between the two age groups. With this aim, we presented older and younger participants semi-artificial simple and complex sentences following a rule of the semi-artificial grammar Japañol: Spanish lexicon with Japanese syntax (see Maie and Dekeyser, 2020 for a similar procedure using English lexicon with Japanese syntax called Japlish). In the incidental condition, participants were presented with the sentences and asked to answer comprehension questions about them, whereas in the intentional condition, they were informed about the rule before being presented with the sentences. The rules appeared in simple (without subordinate clause) or in complex sentences (with subordinate clause). After the learning session, participants were asked to perform a Grammatical Judgment test (GJT) with grammatical and ungrammatical new sentences. These sentences were used to calculate a rule-learning d’ index representing the capacity of the participants to discriminate between grammatical (hits) and ungrammatical (false alarms) new sentences, and therefore, the extent to which participants have learnt the rule. As it was previously found by Midford and Kirsner (2005), we expected better performance in the intentional than in the incidental condition. More importantly, we predicted that younger adults in the intentional conditions would have better performance (higher rule-learning d’) than older adults for both simple and complex sentences, whereas in the incidental conditions, the differences between younger and older adults might not be evident, especially for complex sentences (where procedural strategies are expected).

In addition, to explore the role of individual differences, participants were asked to perform the AX-CPT task (Braver and Barch, 2002) and we calculated the BSI index for each participant (see below for a detailed explanation of how BSI is calculated; Braver, 2012). This index reflects the balance between proactive/reactive control at an individual and group level. As mentioned, previous research has shown differences between younger and older adults in the task with younger adults showing better performance and better proactive control (Braver et al., 2009). Similarly, Rivera et al. (under review) in the context of grammatical learning task, showed that proactive control was positively related to rule-learning in an intentional context where participants were informed of the presence of a regularity (although the particular rule was not explicitly stated). Even though the conditions of the present experiment were different to this previous study (Rivera et al., under review), we hypothesized that proactivity would be related to better intentional learning which requires maintaining the explicit goal to learn the rule in mind (proactive control). We also expected that, overall, older adults would show reduced proactive control as compared to their younger counterparts, and that this, in turn, might be related to reduced performance under intentional conditions. If this was the case, we would also observe that the differences between older and younger adults would diminish for older participants with higher proactive control. Our predictions regarding incidental conditions were less precise since proactive and reactive control might play different roles during incidental learning. On the one hand, proactive control has been related to enhanced responsiveness to contextual cues which might also help to detect language regularities even under incidental conditions. On the other hand, proactive control is cognitively demanding, and it might only be put to work when participants attempt to learn the materials in a motivated and intentional manner and not when participants’ attention is focused on understanding the sentences in the incidental condition. In the latest situation, the less demanding reactive control might be advantageous.

## Materials and methods

### Participants

Sample size for this experiment was calculated *a priori* to estimate the sample. The expected power of fixed-effects was calculated *a priori* using the simr package in R (Brysbaert and Stevens, 2018). The effect-size was planned on a pilot study with 10 participants, and the minimum requirement was estimated through powerCurve function (alpha = 0.4). With 1,000 simulations, the simulation showed a sample size of 134 to achieve 100% statistical power. A total of 162 participants completed the experiment; 81 old adults (*M* = 66; *SD* = 4.7) and 81 young adults (*M* = 21.4; *SD* = 4.8). As a requirement, all of them were native speakers of Spanish with low proficiency in any other second language (B1 or lower according to the European Common Framework), as reported in a self-assessment questionnaire. Participants in each age group were randomly assigned to either the intentional or the incidental learning condition. No differences in age and formal education were found between participants in any of the conditions (all ts < 1; see Table 1). Additionally, to rule out any possible mild cognitive impairments in older adults, we tested them with an online adaptation of the 7 min test (7 MT) (Solomon et al., 1998; Spanish version; Ser Quijano et al., 2004) (from a maximum of 45 points: *M* = 28.59; SD = 8.26; being 22 or less is a sign of decline). In addition, we created a Sociodemographic and Daily Life questionnaire (based on Scarmeas and Stern, 2004), to assess their cognitive reserve (*M* = 2.34; *SD* = 1.18; from a maximum score 5); no differences between conditions of learning, *t* < 1. Overall, our older participants were cognitively active in their daily life. Although the mean cognitive reserve score was medium, 92% of them assured to read in their daily basis. Participants received course credit for their participation, or a raffle ticket for a 25€ card on an online shopping website. All tasks were programmed and ran in Gorilla.sc, an online platform for behavioral experiments (Anwyl-Irvine et al., 2020).

**TABLE 1:** Socio-demographic information extracted from the LEAP-Q questionnaire.

Group	Condition	Age	Formal education (years)	Cognitive reserve
Young	Intentional	22.7 (35.28)	17.58 (3.92)	−
	Incidental	20.1 (1.20)	17.41 (2.07)	−
Old	Intentional	66.68 (4.86)	23.68 (15.75)	2.34 (1.11)
	Incidental	65.3 (4.56)	20.27 (11.16)	2.35 (1.27)

Mean (SD) of age and years of formal education for young and old participants.

### Materials

#### Experimental tasks: Learning and grammaticality judgment test

A total of 100 sentences were generated following two types of rules in a semi-artificial language system. Our semiartificial language Japañol (Spanish lexicon with Japanese syntax) is an adaptation of Japlish (English lexicon with Japanese syntaxis) previously used in other experiments (Williams and Kuribara, 2008). We used the rules used by Maie and Dekeyser (2020) in their simple and complex word order modification. According to these rules, every sentence and clause ends with a verb and there are three case markers depending on grammatical information: -ga for the subject, -o, for the direct object, and -ni for the indirect object. Four different word orders are grammatically correct in Japanese. Two of these word orders were included in simple sentences of the forms: Direct Object-Subject-Verb, (OSV) and Direct Object-Subject-Indirect Object-Verb (OSIV); the other two word orders were included in complex sentences involving subordinate clauses: Direct Object-Subject-(Subject-Verb)-Verb (OSSVV) and Direct Object-Subject-(Subject-Indirect Object-Verb)-Verb (OSSIVV). Twenty-five sentences were generated for each of the four word-orders. From the total of 100 sentences, half of the sentences were plausible. The sentences were randomly presented, and all participants saw all sentences during the training phase (see Supplementary materials for examples).

**FIGURE 1:**
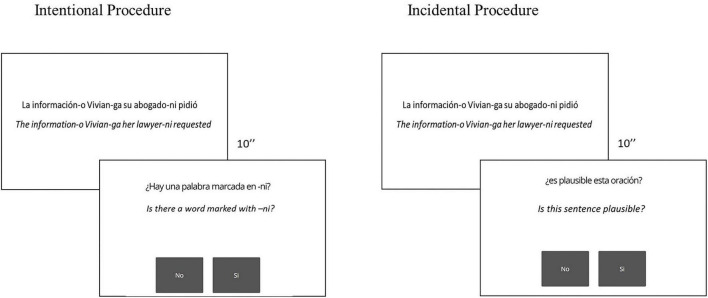
Learning task conditions: Incidental and intentional.

For both intentional and incidental learning contexts, participants were told that Japañol was a South American dialect of Spanish. In the incidental condition, participants were told to read the sentences one by one and respond (yes/no) whether the presented sentence was plausible. They were told that the purpose of this task was to know if the “dialect” was easy to understand for native speakers of Spanish. In the intentional condition, the word order and case marker rules were explicitly explained to the participants before asking them to read the sentences one by one and to respond a question about the presence/absence (yes/no) of a specific feature of the rule after each sentence; half of the sentences had the specific feature asked in the question (participants answered “yes”) and the other half did not have it (participants answered “no”) (see Figure 1). Each sentence appeared on the screen for 10 s and participants responded right after presentations. Although we did not record response times, participants were told to answer as fast and accurate as possible.

Grammaticality Judgment tasks (GJT). After training, participants in the incidental condition were told that the sentences were grammatically correct and that they all followed the rules of the dialect. Additionally, all participants were told that they needed to perform a grammaticality judgment test where they had to respond (yes/no) whether the sentences were grammatically correct. The test encompassed a total of 112 sentences: 32 were previously studied during the training phase (studied; half were plausible); 32 were new sentences that followed the learned rules (new grammatical plausible sentences; all were plausible). For both, studied and new grammatical sentences, the four word-orders representing the rule were equally distributed (eight sentences per word order). Finally, 48 were new sentences that did not follow the rules (new ungrammatical sentences) with eight sentences violating each of the four learned word orders, eight missing a case marker and eight having a case marker changed. All sentences were randomly presented to the participants.

#### AX-Continuous performance task

As mentioned, the AX-CPT tasks has been widely used to assess proactive and reactive control strategies (Locke and Braver, 2008). In this version of the task (Ophir et al., 2009), a set of 5 letters were shown in the middle of the screen following a specific presentation order, the first and the last one were printed in red, and the three middle ones were printed in black. The presentation of the letters created 4 different conditions: (a) AX condition, when the first red letter presented was an “A” and the last red letter presented was an “X,” participants needed to answer “yes”; (b) AY pattern, when the first red letter presented was an “A” but the last red letter presented was not an “X,” the correct answer was “no”; (c) BX pattern, when the first red letter presented was not an “A” but the last red letter presented was an “X,” the correct answer was “no”; (d) BY pattern, when neither the first letter was an “A” nor the last letter was an “X,” the correct answer was “no.” They also had to answer “no” during the middle letters (printed in black). The proportion of the patterns was: 70% for the AX; and 10% for any other pattern (AY, BX, or BY), from a total of 100 trials. This proportion is usually set to induce participants to pay attention to the context since it is highly predictive, and to use proactive control strategies. Participants performed a practice block representing the four experimental conditions where participants were given feedback. After the practice block, they completed the experimental block (100 trials). Participants were asked to answer as fast and accurately as possible. Trials were randomized for each participant. The letters were presented 300 ms in the center of the screen, with 4,900 ms between the presentation of the cue and the probe (printed in red) where the 3 distractor letters (printed in black) were presented for 300 ms with a 1,000 ms interval between them. The interval between trials was 1,000 ms.

### Procedure

Due to restrictions as a result of the COVID-19 pandemic, all tasks were programmed, and the experiment was run using Gorilla.sc, an online platform for behavioral experiments (Anwyl-Irvine et al., 2020). The experiment was divided in two sessions. During the first session, participants learned the rules. As mentioned, in the incidental condition, participants were told to read each sentence and respond if the sentences were or not plausible. In the intentional condition, participants were explicitly informed about the rules before presenting them with the sentences and they were asked to respond (yes/no) whether a specific feature of the rule was in the sentence (see Figure 1). For both intentional and incidental conditions, the sentence remained on the screen for 10 s after a fixation point (300 ms). Then, the question appeared, and remained on the screen until the participants’ response. To respond, participants needed to press the mouse over one of the two boxes that appeared on the screen (yes/no boxes; see Figure 1). Finally, the AX-CPT and the control tasks were presented in the second session. Before each session, participants were contacted by phone to walk them through the Gorilla platform and make sure that if anything went wrong, they would call the researcher.

### Data analysis

#### Grammaticality judgment task

Performance was calculated through discrimination d’ scores (Hautus et al., 2021). The extent to which participants generalized the rule to new sentences was assessed by calculating a d’ index: False Alarms (FA) on new-ungrammatical sentences were subtracted from hits on new-grammatical sentences (Rule-learning d’), indicating more abstract representation of the rule. Secondly, and for sake of completeness, a d’ was calculated for the studied sentences, by subtracting FA on new-ungrammatical sentences from hits on studied-grammatical sentences (Episodic-recognition d’); this represents knowledge of the exact sentences they were trained with. Studied implausible sentences (*n* = 16) were considered as fillers and not included in the analyses. Differences from chance were calculated using one-sample *t*-test between hits and FA (Table 2). Additionally, following signal detection theory (Hautus et al., 2021), we calculated the response criterion index (β) as a measure of response bias. High values of β indicate that participants are using a conservative criterion for “yes” response, whereas lower β-values indicate a more lenient criterion when responding “Yes.”

**TABLE 2:** Mean rates (SD) for *d’* scores and *t*-tests between hits and FA.

Young group				
*d’* score	Incidental condition		Intentional condition	
	Simple	Complex	Simple	Complex
Rule learning	0.88 (1.43)	0.25 (0.70)	2.81 (1.49)	1.74 (1.23)
*T*-test	*t*(43) = 4.57, *p* < 0.001, 95% CI [0.13, 0.34]	*t*(43) = 2.82, *p* = 0.007, 95% CI [0.02, 0.15]	*t*(36) = 12.37, *p* < 0.001, 95% CI [0.59, 0.82]	*t*(36) = 8.92, *p* < 0.001, 95% CI [0.39, 0.63]
Episodic recognition	0.97 (1.42)	0.26(0.69)	2.70 (1.53)	1.80 (1.49)
*T*-test	*t*(43) = 5.06, *p* < 0.001, 95% CI [0.15, 0.37]	*t* (43) = 11.89, *p* = 0.013, 95% CI [0.01, 0.14]	*t* (36) = 11.35, *p* < 0.001, 95% CI [0.57, 0.82]	*t* (36) = 7.43, *p* < 0.001, 95% CI [0.37, 0.65]
**Old group**				
Rule learning	0.36 (0.98)	−0.12 (0.60)	2.21 (1.61)	0.63 (1.11)
*T*-test	*t*(45) = 3.48, *p* = 0.001, 95% CI [0.05, 0.19]	*t*(45) = −0.761, *p* = 0.451, 95% CI [−0.06, 0.02]	*t*(34) = 8.07, *p* < 0.001, 95% CI [0.40, 0.67]	*t*(34) = 3.44, *p* = 0.002, 95% CI [0.07, 0.30]
Episodic recognition	0.38 (0.87)	0.13 (0.64)	2.23 (1.62)	0.53 (1.31)
*T*-test	*t*(45) = 4.38, *p* < 0.001, 95% CI [0.07, 0.20]	*t*(45) = 1.606, *p* = 0.115, 95% CI [−0.01, 0.09]	*t*(34) = 8.221, *p* < 0.001, 95% CI [0.42, 0.71]	*t*(34) = 2.37, *p* = 0.023, 95% CI [0.02, 0.28]

T-test reports for rule-learning d’ and episodic-recognition d’ for young and old adults.

#### AX-Continuous performance task

For the AX-CPT, the data below 100 ms and 2.5 SDs over each participant’s mean were filtered (Zirnstein et al., 2018), for young (5.4%) and old (3.5%) adults. An index was calculated for the AX-CPT task, the Behavioral Shift Index (BSI) was calculated as a combination of AY and BX trials (between errors and Response Time, RT; Braver et al., 2009). The index goes from −1 to + 1, where scores near 0 show a balance between proactive and reactive control (1 more proactive/−1 less proactive).

## Results

First, we analyzed the differences between False Alarms and Hits for each condition to assess overall learning, that is if participants were able to discriminate between grammatical and ungrammatical sentences. Results from the *t*-tests between FA and hits, indicate that young participants discriminated between grammatical (new) and ungrammatical sentences beyond chance, both on simple and complex structures after incidental and intentional learning. However, old adults were not able to discriminate beyond chance on complex structures after incidental learning (see means and *t*-tests on Table 2).

### Rule learning d’ main model

Analyses on rule learning were performed using linear mixed-effects models. We first fitted each model using the automatic function step from the stats-package, version 4.0.0 (R core Team, 2020), specifying direction = “backward.” Thus, the most complex model started with using maximum likelihood (ML). This function removes all meaningless predictors until it finds the model where all factors are statistically significant. The analyses were conducted using the lmer function of the lme4R-package, version 1.1-23 (Bates et al., 2015).

To explore the role of the different factors on rule learning, condition (intentional/incidental), age (young/old), rule-complexity (simple/complex), and BSI (continuous variable) were included in the model as fixed factors. Participants were included as a random factor on the intercept. After fitting the model, the final model contained the interaction for condition and rule-complexity, condition and age, and condition and BSI (see Table 3).

**TABLE 3:** Fixed effects from the LME model of rule learning *d’*.

*Final model*					
Effect	Estimate	SE	*t*	95% CI	*p*
Intercept	–0.24	0.20	–1.21	−0.63, 0.14	0.22
Condition	1.12	0.31	3.61	0.51, 1.72	< 0.001***
Complexity	0.60	0.12	4.80	0.35, 0.84	< 001 ***
Age	0.26	0.23	1.13	−0.19, 0.72	0.25
BSI	1.07	0.61	1.75	−0.12, 2.26	0.08^⋅^
Condition:Complexity	0.71	0.19	3.84	0.35, 1.08	< 0.001***
Condition:Age	0.81	0.35	2.29	0.12, 1.51	0.02*
Condition:BSI	–2.08	0.91	–2.28	−3.88, −0.29	0.02*

*p < 0.05; ***p < 0.001.

**FIGURE 2:**
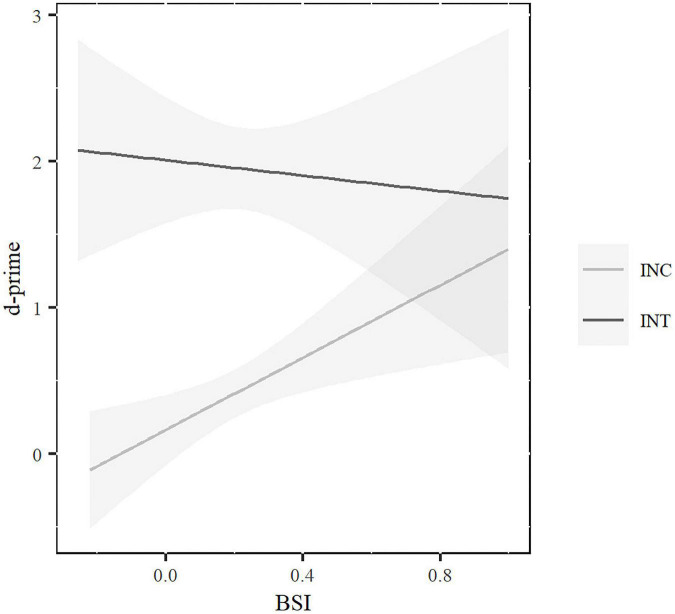
Rule-learning d’ scores associated to BSI for incidental (INC) and intentional (INT) conditions. Highlighted areas represent standard error.

**FIGURE 3:**
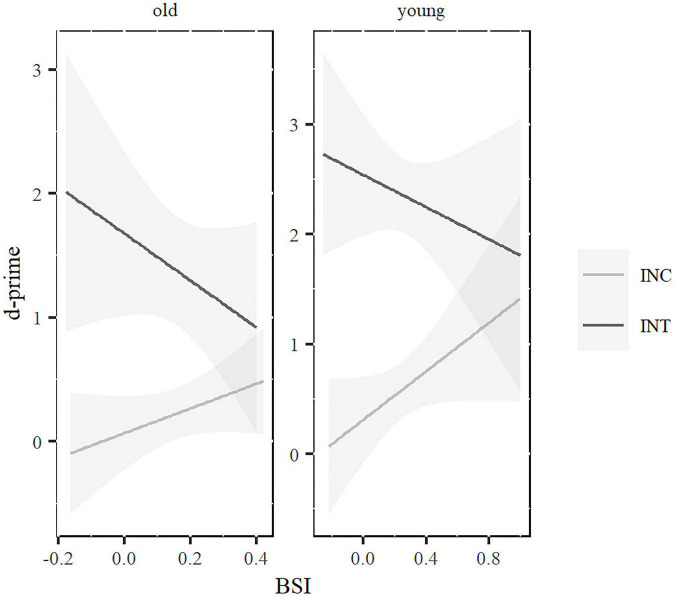
Rule-learning d’ scores associated to BSI for incidental (INC) and intentional (INT) conditions, in younger **(right)** and older **(left)** adults. Highlighted areas represent Standard Error.

Overall, participants showed better performance in the intentional (*M* = 1.95*; SE* = 0.13) than in the incidental condition (*M* = 0.41; *SE* = 0.12) and rules in simple sentences (*M* = 1.56; *SE* = 0.12) were learned better than rules in complex sentences (*M* = 0.64; *SE* = 0.12). No significant main effect of age was found. All significant main effects were modulated by higher level interactions (see Table 4). The learning condition × rule-complexity interaction showed better performance for the simple sentences both in the intentional, *t*(144) = −8.84, *p* < 0.001 and incidental, *t*(144) = −4.11, *p* < 0.001 conditions. However, the differences between simple and complex sentences were larger in the intentional (1.31) than in the incidental (0.6) condition. The learning condition × age interaction showed better performance for young participants than for older adults in the intentional *t*(144) = −3.96, *p* < 0.001, but not in the incidental condition, *t*(144) = −1.11, *p* = 0.68. Crucially, the learning condition was also modulated by a higher interaction with BSI, where the differences between slopes were significant (χ^2^ = 4.98; *p* = 0.02). In the incidental condition, BSI was close to significance, *t*(138) = 1.96 *p* = 0.05. However, no significant significance was found in the intentional condition, *t*(138) = −0.43 *p* = 0.067. As can be seen in Figure 2, larger BSI scores (BSI toward 1) predicted higher d’ learning scores in the incidental condition. As seen in Figure 3, this pattern of results was similar for younger and older participants since the three-way interaction with age was not significant.

**TABLE 4:** *d’* mean and standard deviation per condition of learning, age group, and complexity.

Intentional condition			
	Young	Old	Overall
Simple	2.9 (0.19)	2.16 (0.22)	2.6 (0.15)
Complex	1.77 (0.19)	0.57 (0.22)	1.29 (0.15)
Overall	2.34 (0.17)	1.38 (0.21)	
**Incidental condition**			
Simple	0.90 (0.18)	0.49 (0.19)	0.71 (0.14)
Complex	0.26 (0.18)	−0.05 (0.19)	0.11 (0.14)
Overall	0.58 (0.15)	0.22 (0.17)	

### Rule learning β main model

No significant main effects of condition, age, complexity, or BSI were found. However, the three-way interaction between learning condition × age × complexity (see Table 5) showed that in the intentional condition, the younger group has a more conservative criterion (*M* = 7.99; SD = 0.95) than the older group (*M* = 2.08; SD = 1.16) when learning the more difficult sentences *t*(62) = 3.8, *p* = 0.001. In contrast, no differences were found between younger and older participants for the incidental condition *t*(148) = −1.12, *p* = 0.67. When learning simple sentences, no significant differences were found for the intentional *t*(63) = −0.18, *p* = 0.99 nor the incidental *t*(148) = −0.54, *p* = 0.94 condition.

**TABLE 5:** Fixed effects from the LME model of rule learning β.

Effect	Estimate	*SE*	*t*	95% CI	*P*
**Final model**					
Intercept	0.17	0.66	0.26	−1.12, 1.47	0.79
Condition	1.91	1.04	1.83	−0.12, 3.95	0.07
Complexity	–0.37	0.89	–0.42	−2.11, 1.37	0.68
Age	0.42	0.91	0.47	−1.35, 2.21	0.67
Condition:Complexity	–1.49	1.39	–1.07	−4.2, 1.22	0.28
Condition:Age	5.48	1.38	3.97	2.77, 8.18	<0.001***
Complexity:Age	–0.22	1.22	–0.18	−2.61, 2.16	0.85
Condition:Complexity:Age	–5.41	0.84	–2.94	−9.01, −0.18	0.003**

*p < 0.05; **p < 0.01; ***p < 0.001.

## Discussion

Second language grammar learning is a big challenge, especially for the elderly. Cognitive decline seems to predict differences in the learning strategies of younger and older adults (Marcotte and Ansaldo, 2014). However, these differences seem to be less evident during incidental learning processes (Midford and Kirsner, 2005). In the present experiment, we aimed to explore the different learning strategies of older and younger adults when learning a new (semi-artificial) grammar, and how extrinsic (learning conditions and complexity of the learning material) and intrinsic (cognitive control) factors affected these strategies.

Altogether, the results of the experiment indicated that both younger and older participants obtained better learning of the semi-artificial grammar under intentional learning conditions than under incidental learning conditions. The advantage on intentional learning goes in line with what it has been previously reported in the second language learning literature (see Goo et al., 2015 for a review). More importantly, this main effect was modulated by a high order interaction with age, indicating that younger adults were better learners than older adults just in the intentional condition of learning. No differences between age groups were found when learning in an incidental condition where both groups acquired the semi-artificial grammar to similar levels. These results are in line with Midford and Kirsner (2005) who reported better intentional grammar learning for younger than older adults, and similar lower levels of learning for younger and older adults under incidental learning conditions (see Verneau et al., 2014 for similar results in a motor skill acquisition task). Interestingly, they also found that there were not differences between intentional and incidental conditions for older adults, suggesting that they might have used similar learning strategies with and without metalinguistic instructions. However, and differently from Midford and Kirsner (2005), we found that older adults were still able to take advantage of the metalinguistic information provided in the intentional condition since the intentional advantage was still evident in older adults despite their performance was significantly lower than that for younger adults. Although, the reasons for this discrepancy need to be further explored, it is possible that the higher level of education and cognitive reserve of our participants as compared to Midford and Kirsner (2005) may underlie the intentional advantage displayed by our older participants. As a matter of fact, while older adults in our study had 22 years of formal education in average, older adults in Mildford and Kirsner’s study had 13. In addition, most of our participants came from university courses, 92% of them reported to read in a daily basis and all were able to use a computer and internet independently (as it was the main reason to join the experiment). Therefore, as evidenced before in the literature, the cognitive strategies in older adults can be preserved thanks to high cognitive reserve (Stern, 2009).

In the same line, the complexity of the sentences affected language learning similarly to younger and older adults. Thus, rules in simple sentences were better learned than rules in complex sentences, but the difference between simple and complex sentences was similar for both groups. In addition, complexity particularly affected intentional learning as suggested by the significant interaction between complexity and learning condition, but again this interaction was similar for younger and older adults, suggesting that our older participants were able to cope with some of the difficulties imposed by the task. Interestingly, significant differences were found between younger and older adults for β (response criterion) in the intentional learning condition. Younger adults had a more conservative criterion than the older adults when answering to difficult sentences, however, there were no differences between age groups in d’. Hence, we can assume that there are differences in the response strategies used by younger and older adults. Thus, although older and younger participants did not differ in learning success, younger adults were able to adjust their strategies and respond more cautiously when confronted with more difficult sentences.

In addition, we wanted to explore how individual differences in cognitive control could affect grammar learning and if it did differently for younger and older adults. As mentioned, we expected to find proactivity highly related to intentional learning success (Rivera et al., under review). We also expected that the differences between older and younger adults in intentional learning would be less evident for older participants with higher proactive control. However, this was not the case. Although young participants showed higher BSI than older adults indicating that they were more proactive, proactivity did not have any significant effect in intentional learning for either of the two groups, suggesting that participants were able to hold the task goals (detect features of the rule) in mind independently of their proactive control. Further research should be directed to assess if more complex task goals in the intentional condition might benefit from proactive control (i.e., using unknown lexicon).

In contrast, the significant interaction between BSI and condition of learning showed that, in incidental learning, BSI was positive related to learning success: participants with high proactive control (BSI toward 1) were significantly better than participants with low proactive control (BSI toward −1). Proactive control involves monitoring the environment to prepare for the task goals, which in turn may facilitate the detection of language regularities that might be critical for incidental learning. Our results suggests that the context monitoring and task readiness involved in proactive control helps participants to unconsciously learn something based on the regularities of the material. It is the first study to find the implication of proactive control in the process of incidental learning. Contrary to the hypothesis talking about the benefits of “learning without control” (Thompson-Schill et al., 2009), we found that the presence of proactive control is involved in the grammatical learning process, at least, in the incidental conditions of our experiment. In line with the “more is more” hypothesis (Brooks and Kempe, 2019), perhaps the existence of stronger cognitive abilities can facilitate the acquisition of new grammatical rules at younger and older ages. Thus, learners with stronger proactive skills seem to benefit from the mere exposure to the grammatical sentences. However, since this is the first experiment on the role of proactive control on incidental learning, more research is needed to draw strong conclusions about this relationship, particularly since the online nature of our experiment (due to COVID restrictions) influenced the composition of the sample “filtering out” those older adults without computer skills. It is possible that a similar experiment in a laboratory setting would yield different results. Future research needs to consider the extra implications of online experimentation for target populations other than young adults who, in average, use technological devices in their daily life. Additionally, it would also be interesting to study the role of other variables associated to metalinguistic awareness (Li and DeKeyser, 2021 for a review) or motivation to learn (Dörnyei, 2020) as modulatory factors on our results.

To summarize, we found that younger adults are better learners under intentional conditions than older adults, although older adults were able to take advantage of the metalinguistic information provided by the instruction in the intentional condition as they showed better performance for intentional than incidental conditions. No age differences were found under incidental conditions. Additionally, we found that, for both age groups intentional learning was not affected by executive control, but proactivity benefited incidental learning of the grammatical rules. Therefore, we can conclude that individual differences in cognitive control affect in the same way to both age groups, since the older group had similar cognitive strategies during the learning process. However, more research is needed to properly understand where these learning differences come from, which cognitive factors are key, and which is the real implication of cognitive reserve in the relation between grammar learning and cognitive strategies.

## Data availability statement

The raw data supporting the conclusions of this article will be made available by the authors, without undue reservation.

## Ethics statement

The studies involving human participants were reviewed and approved by the Ethical Committee of the University of Granada (1722/CEIH/2020). The patients/participants provided their written informed consent to participate in this study.

## Author contributions

All authors listed have made a substantial, direct, and intellectual contribution to the work, and approved it for publication.
