# Supplementary material for: Cognitive and contextual factors modulating grammar learning at older ages
Source: Front Aging Neurosci. 2022 Aug 31;14:943392. doi: 10.3389/fnagi.2022.943392 (PMC9471146; doi:10.3389/fnagi.2022.943392)
Supplement: Supplementary file 1 [file Table_1.DOCX]

Supplementary Material

**Learning Material**

| **WORD ORDER** | **SENTENCE** |
| --- | --- |
| O-S-V | Esta bicicleta-o Jose-ga compró |
| O-S-V | Esta pared-o María-ga pintó |
| O-S-V | La tarta-o Miguel-ga comió |
| O-S-V | La tinta-o Estela-ga derramó |
| O-S-V | Esas canciones-o Jaime-ga cantó |
| O-S-V | La puerta-o Carmen-ga rompió |
| O-S-V | Un ejercicio-o Tomás-ga terminó |
| O-S-V | El fuego-o Angela-ga encendió |
| O-S-V | Ese bolígrafo-o Tomás-ga usó |
| O-S-V | El vídeo Carlos-ga vió |
| O-S-V | Esta silla-o Marta-ga llevó |
| O-S-V | Una llave-o Linda-ga tiró |
| O-S-V | Esas palabras-go Esteban-ga aprendió |
| O-S-V | Una puerta-o Tomás-ga bebió |
| O-S-V | El plato-o Carlos-ga habló |
| O-S-V | La niña-o Marta-ga construyó |
| O-S-V | Un móvil-o Linda-ga escribió |
| O-S-V | Las escuelas-o Esteban-ga jugó |
| O-S-V | Esos zapatos-o Pamela-ga comió |
| O-S-V | El libro-o Jorge-ga respiró |
| O-S-V | Una habitación-o Nicolás-ga previnió |
| O-S-V | La guitarra-o Jose-ga peló |
| O-S-V | Un año-o María-ga movió |
| O-S-V | Una carta-o Miguel-ga ofendió |
| O-S-V | Este cubo-o Estela-ga calmó |
| O-S-I-V | El cuadro-o Jose-ga sus amigos-ni envió |
| O-S-I-V | Una carta-o María-ga su jefe-ni mandó |
| O-S-I-V | Este idioma-o Miguel-ga sus estudiantes-ni enseñó |
| O-S-I-V | Un jersey-o Estela-ga su marido-ni regaló |
| O-S-I-V | Su licencia-o Jaime-ga la policia-ni mostró |
| O-S-I-V | La sal-o Carmen-ga su hermano-ni pasó |
| O-S-I-V | Un hueso-o Tomás-ga el perro-ni dío |
| O-S-I-V | Paz-o Angela-ga el gobierno-ni demandó |
| O-S-I-V | Un restaurante-o Tomás-ga sus padres-ni recomendó |
| O-S-I-V | Una carta-o Carlos-ga su novio-ni escribió |
| O-S-I-V | Este regalo-o Marta-ga su amigo-ni compró |
| O-S-I-V | Algún dinero-o Linda-ga un banco-ni depostió |
| O-S-I-V | Ese ordenador-o Esteban-ga su hijo-ni compró |
| O-S-I-V | Una pregunta-o Tomás-ga el libro-ni hizo |
| O-S-I-V | Algún dinero-o Carlos-ga un gato-ni dejó |
| O-S-I-V | El secreto-o Marta-ga esta mesa-ni contó |
| O-S-I-V | Un trabajo-o Linda-ga ese cohete-ni ofreció |
| O-S-I-V | Una pelota-o Esteban-ga una piedra-ni lanzó |
| O-S-I-V | Esta galleta-o Pamela-ga su bolsa-ni horneó |
| O-S-I-V | La medalla-o Jorge-ga la toalla-ni ganó |
| O-S-I-V | Una canción-o Nicolás-ga un impuesto-ni cantó |
| O-S-I-V | Una historia-o Jose-ga la ciudad-ni recitó |
| O-S-I-V | Un trato-o María-ga un virus-ni propuso |
| O-S-I-V | Esta flor-o Miguel-ga la cualidad-ni compró |
| O-S-I-V | Esta comida-o Estela-ga una escalera-ni alimentó |
| O-S-[S-V]-V | El diamante-o Jose-ga este hombre-ga había robado pensó |
| O-S-[S-V]-V | La enseñanza-o María-ga su colegio-ga había crecido mencionó |
| O-S-[S-V]-V | Los donuts-o Miguel-ga su perro-ga había comido descubrió |
| O-S-[S-V]-V | Ese vaso-o Estela-ga su esposa-ga había roto dijo |
| O-S-[S-V]-V | Su coche-o Jaime-ga un cilcista-ga había dañado pensó |
| O-S-[S-V]-V | Esa niña-o Karen-ga la policia-ga había encontrado mencionó |
| O-S-[S-V]-V | Su salud-o Tomás-ga el fumar-ga había dañado descubrió |
| O-S-[S-V]-V | Una pistola-o Angela-ga un ladrón-ga había disparado dijo |
| O-S-[S-V]-V | Alguna leche-o Tomás-ga su madre-ga había añadido pensó |
| O-S-[S-V]-V | El gobernador-o Carlos-ga su compañero-ga había abusado mencionó |
| O-S-[S-V]-V | Esos niños-o Marta-ga su amigo-ga había asustado descubrió |
| O-S-[S-V]-V | Ese sushi-o Linda-ga ese chef-ga había hecho dijo |
| O-S-[S-V]-V | El trabajo-o Esteban-ga su padre-ga había dejado pensó |
| O-S-[S-V]-V | Un hicapié-o Tomás-ga el tren-ga había golpeado mencionó |
| O-S-[S-V]-V | El teatro-o Carlos-ga un cantante-ga había tocado descubrió |
| O-S-[S-V]-V | Una habilidad-o Marta-ga el fuego-ga había quemado dijo |
| O-S-[S-V]-V | Un desenlace-o Linda-ga su jefe-ga había despidido pensó |
| O-S-[S-V]-V | Esos libros-o Estaban-ga la camarera-ga había derramado mencionó |
| O-S-[S-V]-V | Este artículo-o Pamela-ga su compañera-ga había conducido descubrió |
| O-S-[S-V]-V | Esas plantas-o Jorge-ga el niño-ga había bebido dijo |
| O-S-[S-V]-V | Esas noticias-o Nicolás-ga su profesora-ga había regañado pensó |
| O-S-[S-V]-V | Una sabana-o Jose-ga su primo-ga había volado mencionó |
| O-S-[S-V]-V | Un libro-o María-ga un elefante-ga había bebido realizó |
| O-S-[S-V]-V | Esta lámpara-o Miguel-ga su maestro-ga había empezado dijo |
| O-S-[S-V]-V | El miedo-o Estela-ga su hija-ga había cargado pensó |
| O-S-[S-I-V]-V | Alguna comida-o Jose-ga su mujer-ga su perro-ni había cogido pensó |
| O-S-[S-I-V]-V | Los documentos-o Maria-ga su compañero-ga su jefe había enviado mencionó |
| O-S-[S-I-V]-V | Este café-o Miguel-ga un estudiante-ga el profesor-ni había cogido descubrió |
| O-S-[S-I-V]-V | Un regalo-o Estela-ga el niño-ga su madre-ni había enviado dijo |
| O-S-[S-I-V]-V | El libro-o Jaime-ga su hermana-ga su amiga-ni había enviado pensó |
| O-S-[S-I-V]-V | Un soborno-o Carmen-ga su compañera-ga su jefe-ni había ofrecido mencionó |
| O-S-[S-I-V]-V | Un consejo-o Tomás-ga su amigo-ga el conductor-ni había dado descubrió |
| O-S-[S-I-V]-V | Una carta-o Angela-ga su marido-ga el alcalde-ni había escrito dijo |
| O-S-[S-I-V]-V | Un collar-o Tomás-ga su amigo-ga su mujer-ni había dado pensó |
| O-S-[S-I-V]-V | Ese coche-o Carlos-ga su mujer-ga el vecino-ni había vendido mencionó |
| O-S-[S-I-V]-V | El secreto-o Linda-ga su novio-ga su madre-ni había contado descubrió |
| O-S-[S-I-V]-V | Algun dinero-o Marta-ga su tio-ga un hombre-ni había salvado dijo |
| O-S-[S-I-V]-V | La pizza-o Esteban-ga su amigo-ga un compañero-ni había guadado pensó |
| O-S-[S-I-V]-V | Un coche-o Tomas-ga sus padres-ga la belleza-ni había comprado mencionó |
| O-S-[S-I-V]-V | Una bicicleta-o Carmen-ga su tia-ga un sabor-ni había presentado descubrió |
| O-S-[S-I-V]-V | Una camarera-o Marta-ga su marido-ga su hijo-ni había leído dijo |
| O-S-[S-I-V]-V | Una medalla-o Linda-ga el presidente-ga el futuro-ni había premiado pensó |
| O-S-[S-I-V]-V | Un honor-o Pamela-ga el rey-ga un descanso-ni había otorgado dijo |
| O-S-[S-I-V]-V | La pena-o Esteban-ga su manager-ga su amigo-ni había propuesto descubrió |
| O-S-[S-I-V]-V | Una miseria-o Jorge-ga el estudiante-ga su maestro-ni había preguntado dijo |
| O-S-[S-I-V]-V | Una victoria-o Nicolas-ga su padre-ga la policia-ni había mostrado pensó |
| O-S-[S-I-V]-V | Un número-o Jose-ga la camarera-ga un cliente-ni había ofrecido mencionó |
| O-S-[S-I-V]-V | Una carta-o María-ga su compañer-ga la paciencia-ni había escrito descubrió |
| O-S-[S-I-V]-V | El libro-o Miguel-ga su profesor-ga el movimiento-ni había dejado dijo |
| O-S-[S-I-V]-V | Un conflicto-o Estela-ga su marido-ga su hijo-ni había comprado pensó |

**Grammatical Judgment Test Material**

| **WORD ORDER** | **SENTENCE** | **GRAMMATICALITY** |
| --- | --- | --- |
| O-S-V | Esta tarta-o Daniel-ga dió | GRAMATICAL |
| O-S-V | La mesa-o Sara-ga limpió | GRAMATICAL |
| O-S-V | La admisión-o Nico-ga aseguró | GRAMATICAL |
| O-S-V | Un paquete-o Emma-ga recibió | GRAMATICAL |
| O-S-V | La calle-o Cristina-ga cruzó | GRAMATICAL |
| O-S-V | Estas lecciones-o Alicia-ga estudió | GRAMATICAL |
| O-S-V | Un perro-o Lola- tuvo | GRAMATICAL |
| O-S-V | El estilo-o Raquel-ga cambió | GRAMATICAL |
| O-S-I-V | La solicitud-o Bruno-ga una universidad-ni mandó | GRAMATICAL |
| O-S-I-V | Una fiesta-o Vivian-ga su madre-ni tiró | GRAMATICAL |
| O-S-I-V | Una lección-o Ricardo-ga sus estudiantes-ni dio | GRAMATICAL |
| O-S-I-V | Esa pelota-o Elisa-ga un jugador-ni arrojó | GRAMATICAL |
| O-S-I-V | Un autógrafo-o Elvis-ga su fan-ni firmó | GRAMATICAL |
| O-S-I-V | Este ordenador-o Desiré-ga su padre-ni eligió | GRAMATICAL |
| O-S-I-V | Ese dinero-o Antonio-ga una iglesia donó | GRAMATICAL |
| O-S-I-V | La verdad-o Lila-ga su profesor-ni dijo | GRAMATICAL |
| O-S-[S-V]-V | El examen-o Benito-ga su amigo-ga había pasado dijo | GRAMATICAL |
| O-S-[S-V]-V | Estos juguetes-o Clara-ga su hijo-ga quería descubrió | GRAMATICAL |
| O-S-[S-V]-V | Esos niños-o Boris-ga ese hombre-ga había ayudado mencionó | GRAMATICAL |
| O-S-[S-V]-V | Su corazón-o Lidia-ga su novio-ga había destruido pensó | GRAMATICAL |
| O-S-[S-V]-V | La guerra-o Óscar-ga los soldados-ga habían sobrevivido dijo | GRAMATICAL |
| O-S-[S-V]-V | El profesor-o Laura-ga su amiga-ga había enfadado descubrió | GRAMATICAL |
| O-S-[S-V]-V | Su artículo-o Guillermo-ga la revista-ga había aceptado mencionó | GRAMATICAL |
| O-S-[S-V]-V | Esos estudiantes-o Patricia-ga la decana-ga había advertido pensó | GRAMATICAL |
| O-S-[S-I-V]-V | Una historia-o Felipe-ga su mujer-ga su hijo-ni había narrado dijo | GRAMATICAL |
| O-S-[S-I-V]-V | Un cheque-o Alba-ga su amiga-ga el vendedor-ni había escrito descubrió | GRAMATICAL |
| O-S-[S-I-V]-V | La ley-o Andrés-ga el congreso-ga el presidente-ni había propuesto mencionó | GRAMATICAL |
| O-S-[S-I-V]-V | Este documento-o Julia-ga su amigo-ga su jefe-ni había mandado pensó | GRAMATICAL |
| O-S-[S-I-V]-V | Una bufanda-o Eric-ga su hermano-ga su madre-ni había dado dijo | GRAMATICAL |
| O-S-[S-I-V]-V | Las flores-o Nuria-ga su vecino-ga su hermana-ni había mandado dijo | GRAMATICAL |
| O-S-[S-I-V]-V | Un trato-o Borja-ga su compañero-ga un cliente-ni había ofrecido mencionó | GRAMATICAL |
| O-S-[S-I-V]-V | Su secreto-o Olivia-ga su hermano-ga sus padres-ni había dicho pensó | GRAMATICAL |
| O !V S | Un coche-o limpió Daniel-ga | UNGRAMMATICAL |
| O !V S | Un barco-o llevó Sara-ga | UNGRAMMATICAL |
| O !V S | La cena-o cocinó Nico-ga | UNGRAMMATICAL |
| O !V S | El bebé-o alimentó Emma-ga | UNGRAMMATICAL |
| O !V S | Una escalera-o subió Cristina-ga | UNGRAMMATICAL |
| O !V S | Este ordenador-o usó Alison-ga | UNGRAMMATICAL |
| O !V S | La radio-o arregló Benito-ga | UNGRAMMATICAL |
| O !V S | Una manzana-o peló Raquel-ga | UNGRAMMATICAL |
| O S V !I | Un café-o Bruno-ga elaboró su mujer-ni | UNGRAMMATICAL |
| O S V !I | Un email-o Vivian-ga reenvió su compañera-ni | UNGRAMMATICAL |
| O S V !I | El beneficio-o Ricardo-ga compartió sus empleados-ni | UNGRAMMATICAL |
| O S V !I | El patrimonio-o Elisa-ga dejó sus hijos-ni | UNGRAMMATICAL |
| O S V !I | Un dólar-o Elvis-ga pagó el cajero-ni | UNGRAMMATICAL |
| O S V !I | Una nana-o Desiré-ga cantó su hijo-ni | UNGRAMMATICAL |
| O S V !I | Algún dinero-o Antonio-ga apostó su amigo-ni | UNGRAMMATICAL |
| O S V !I | Un vestido-o Lila-ga hizo su hija-ni | UNGRAMMATICAL |
| O S V !S V | La promesa-Benito-ga había roto su mujer-ga dijo | UNGRAMMATICAL |
| O S V !S V | Un vaso-o Clara-ga había roto su hijo-ga descubrió | UNGRAMMATICAL |
| O S V !S V | Esos hombres-o Boris-ga había parado la policía-ga mencionó | UNGRAMMATICAL |
| O S V !S V | El árbol-o Lidia-ga había decorado su padre-ga pensó | UNGRAMMATICAL |
| O S V !S V | Un monstruo-o Pablo-ga había realizado el capitán-ga dijo | UNGRAMMATICAL |
| O S V !S V | Ese ordenador-o Laura-ga arregó su novio-ga descubrió | UNGRAMMATICAL |
| O S V !S V | El país Guillermo-ga había liberado los militares-ga mencionó | UNGRAMMATICAL |
| O S V !S V | Cinco idiomas-o Patricia-ga hablaba su profesor-ga pensó | UNGRAMMATICAL |
| O S S V !I V | Un consejo-o Felipe-ga su mujer-ga había dado niño-ni dijo | UNGRAMMATICAL |
| O S S V !I V | Esas heridas-o Alba-ga su marido-ga había enseñado un doctor-ni descubrió | UNGRAMMATICAL |
| O S S V !I V | Las notas-o Alicia-ga su profesor-ga había reportado el director-ni mencionó | UNGRAMMATICAL |
| O S S V !I V | Un coche-o Julia-ga sus padres-ga habían prestado su hijo pensó | UNGRAMMATICAL |
| O S S V !I V | Su duda-o Eric-ga su hermano-ga había pagado el banco-si dijo | UNGRAMMATICAL |
| O S S V !I V | Sus sintomas-o Nora-ga su hermana-ga había explicado una enfermera-ni descubrió | UNGRAMMATICAL |
| O S S V !I V | Un iphone-o Bruno-ga sus padres-ga había regalado su hermano-ni mencionó | UNGRAMMATICAL |
| O S S V !I V | Un acceso-o Olivia-ga el gobernador-ga había concedido su compañero-ni pensó | UNGRAMMATICAL |
| Case Missing | La información-o Daniel su compañero-ni dio | UNGRAMMATICAL |
| Case Missing | La regla Sara-ga su amiga-ni describió | UNGRAMMATICAL |
| Case Missing | Una bicicleta-o Nico-ga su hijo compró | UNGRAMMATICAL |
| Case Missing | Un éxito-o Emma su novio-ni deseó | UNGRAMMATICAL |
| Case Missing | Esta libreta Cristina-ga su hemana-ni devolvió | UNGRAMMATICAL |
| Case Missing | Su traducción-o Alison-ga sus padres enseñó | UNGRAMMATICAL |
| Case Missing | Un plan-o Benito su jefe-ni ofreció | UNGRAMMATICAL |
| Case Missing | El incidente Raquel-ga su vecina-ni describió | UNGRAMMATICAL |
| Case Missing | Una invitación-ga Bruno-o sus compañeros-ni envió | UNGRAMMATICAL |
| Case Missing | La injección-o Vivian-ni su perro-ga dio | UNGRAMMATICAL |
| Case Missing | Un paquete-ni Ricardo-ga sus padres-o dejó | UNGRAMMATICAL |
| Case Missing | Una reforma-ga Elisa-o el ayuntamiento-ni demandó | UNGRAMMATICAL |
| Case Missing | Su tiempo-o Elvis-ni su hijo-ga dedicó | UNGRAMMATICAL |
| Case Missing | Dos entradas-ni Desiré-ga sus padres-o reservó | UNGRAMMATICAL |
| Case Missing | Un café-ga Antonio-o su cliente-ni preparó | UNGRAMMATICAL |
| Case Missing | Una baguette-o Lila-ni su marido-ga horneó | UNGRAMMATICAL |
| O-S-V | Esta bicicleta-o Jose-ga compró | STUDIED |
| O-S-V | Esta pared-o María-ga pintó | STUDIED |
| O-S-V | La tarta-o Miguel-ga comió | STUDIED |
| O-S-V | La tinta-o Estela-ga derramó | STUDIED |
| O-S-V | Una puerta-o Tomás-ga bebió | STUDIED |
| O-S-V | El plato-o Carlos-ga habló | STUDIED |
| O-S-V | La niña-o Marta-ga construyó | STUDIED |
| O-S-V | Un móvil-o Linda-ga escribió | STUDIED |
| O-S-I-V | El cuadro-o Jose-ga sus amigos-ni envió | STUDIED |
| O-S-I-V | Una carta-o María-ga su jefe-ni mandó | STUDIED |
| O-S-I-V | Este idioma-o Miguel-ga sus estudiantes-ni enseñó | STUDIED |
| O-S-I-V | Un jersey-o Estela-ga su marido-ni regaló | STUDIED |
| O-S-I-V | Una pelota-o Esteban-ga una piedra-ni lanzó | STUDIED |
| O-S-I-V | Esta galleta-o Pamela-ga su bolsa-ni horneó | STUDIED |
| O-S-I-V | La medalla-o Jorge-ga la toalla-ni ganó | STUDIED |
| O-S-I-V | Una canción-o Nicolás-ga un impuesto-ni cantó | STUDIED |
| O-S-[S-V]-V | El diamante-o Jose-ga este hombre-ga había robado pensó | STUDIED |
| O-S-[S-V]-V | La enseñanza-o María-ga su colegio-ga había crecido mencionó | STUDIED |
| O-S-[S-V]-V | Los donuts-o Miguel-ga su perro-ga había comido descubrió | STUDIED |
| O-S-[S-V]-V | Ese vaso-o Estela-ga su esposa-ga había roto dijo | STUDIED |
| O-S-[S-V]-V | Un hicapié-o Tomás-ga el tren-ga había golpeado mencionó | STUDIED |
| O-S-[S-V]-V | El teatro-o Carlos-ga un cantante-ga había tocado descubrió | STUDIED |
| O-S-[S-V]-V | Una habilidad-o Marta-ga el fuego-ga había quemado dijo | STUDIED |
| O-S-[S-V]-V | Un desenlace-o Linda-ga su jefe-ga había despidido pensó | STUDIED |
| O-S-[S-I-V]-V | Alguna comida-o Jose-ga su mujer-ga su perro-ni había cogido pensó | STUDIED |
| O-S-[S-I-V]-V | Los documentos-o Maria-ga su compañero-ga su jefe había enviado mencionó | STUDIED |
| O-S-[S-I-V]-V | Este café-o Miguel-ga un estudiante-ga el profesor-ni había cogido descubrió | STUDIED |
| O-S-[S-I-V]-V | Un regalo-o Estela-ga el niño-ga su madre-ni había enviado dijo | STUDIED |
| O-S-[S-I-V]-V | Un coche-o Tomas-ga sus padres-ga la belleza-ni había comprado mencionó | STUDIED |
| O-S-[S-I-V]-V | Una bicicleta-o Carmen-ga su tia-ga un sabor-ni había presentado descubrió | STUDIED |
| O-S-[S-I-V]-V | Una camarera-o Marta-ga su marido-ga su hijo-ni había leído dijo | STUDIED |
| O-S-[S-I-V]-V | Una medalla-o Linda-ga el presidente-ga el futuro-ni había premiado pensó | STUDIED |
